# Assessing Seroprevalence and Infection Dynamics of Oncogenic Gammaherpesviruses in South African Paediatric Patients Presenting with Inflammatory Conditions

**DOI:** 10.3390/ijms27031275

**Published:** 2026-01-27

**Authors:** Katrin Bratl, Claire Butters, Kate Webb, Georgia Schäfer

**Affiliations:** 1 International Centre for Genetic Engineering and Biotechnology (ICGEB), Cape Town 7925, South Africa; brtkat011@myuct.ac.za; 2 Institute of Infectious Disease and Molecular Medicine (IDM), Faculty of Health Sciences, University of Cape Town, Cape Town 7925, South Africa; 3 Division of Medical Biochemistry and Structural Biology, Department of Integrative Biomedical Sciences, Faculty of Health Sciences, University of Cape Town, Cape Town 7925, South Africa; 4 Division of Rheumatology, Department of Paediatrics and Child Health, University of Cape Town, Cape Town 7700, South Africa; chulsman@sun.ac.za (C.B.); kate.webb@uct.ac.za (K.W.); 5 Francis Crick Institute, London NW1 1AT, UK; 6 Wellcome Centre for Infectious Diseases Research in Africa, University of Cape Town, Cape Town 7925, South Africa

**Keywords:** Kaposi’s sarcoma-associated herpesvirus (KSHV or HHV-8), Epstein–Barr virus (EBV or HHV-4), severe acute respiratory syndrome coronavirus 2 (SARS-CoV-2), COVID-19, Kawasaki disease (KD), multisystem inflammatory syndrome in children (MIS-C), Sub-Saharan Africa

## Abstract

Kaposi’s Sarcoma-associated herpesvirus (KSHV) and Epstein–Barr virus (EBV) are oncogenic gammaherpesviruses with high prevalence in sub-Saharan Africa. Both viruses are typically acquired during childhood, establishing lifelong latency. While viral reactivation into the lytic cycle has been mainly studied in adult HIV-infected populations—and more recently in the context of severe acute respiratory syndrome coronavirus 2 (SARS-CoV-2) co-infection—the dynamics of KSHV and EBV infection in children remain poorly understood. Here, we characterize pediatric patients (n = 175; median age 4.6 years; IQR 2.0–8.3) presenting with inflammatory conditions during the COVID-19 pandemic in South Africa (from July 2020 to February 2024). Including a healthy, non-inflammatory control group, we found widespread exposure to SARS-CoV-2 (70.9% seropositivity), with 72.6% of the children being seropositive for EBV and 19.4% for KSHV. There were no significant differences in seroprevalence between children with inflammatory conditions and healthy controls for any of these viruses, although SARS-CoV-2 antibody titers were significantly higher in the inflammatory group, while EBV immune responses were lower in children presenting with inflammation. Among the KSHV-seropositive children, no active viremia was detected (as determined by the absence of viral DNA in the peripheral blood). In contrast, 34.4% of EBV-seropositive children had detectable EBV viral load, with a modestly higher proportion in the inflammatory group. However, EBV viral load levels were comparable between children diagnosed with Multisystem Inflammatory Syndrome in Children (MIS-C), Kawasaki Disease (KD), and other inflammatory conditions. Logistic regression analyses revealed that increasing age was significantly associated with higher risk of SARS-CoV-2 and EBV seropositivity, but not KSHV. Notably, the risk of EBV DNA detection in the peripheral blood decreased with age. In summary, this study suggests effective immunological control of gammaherpesvirus infections in HIV-negative paediatric patients, even in the presence of inflammatory conditions that might otherwise trigger viral reactivation.

## 1. Introduction

Epstein-Barr virus (EBV) and Kaposi’s sarcoma-associated herpesvirus (KSHV) are human oncogenic herpesviruses belonging to the subfamily Gammaherpesvirinae of the Herpesviridae family. Human herpesviruses are a large group of enveloped, double-stranded DNA viruses, characterized by their ability to establish lifelong infections within their hosts [1]. Both EBV and KSHV are capable of manipulating host cell signalling and evading immune responses, ultimately allowing them to persist in the host and, potentially, contribute to tumorigenesis. This is particularly relevant in individuals with Human Immunodeficiency Virus (HIV)-related immunosuppression, with Kaposi’s Sarcoma (KS) being the most common AIDS-related malignancy worldwide [2]. While EBV infection is ubiquitous with a prevalence of over 90% globally, KSHV prevalence varies geographically, with seroprevalence rates of less than 10% reported in Europe and the United States and between 50 and 75% in Sub-Saharan Africa (SSA), where KSHV is endemic [3,4,5,6,7,8,9].

Transmission of EBV and KSHV primarily occurs in childhood via saliva from mother to child or between siblings [10]; however, sexual contact and blood or blood products have also been described as transmission routes [11,12,13,14]. Upon acute infection, both viruses establish latency in the host, which can be interrupted by intermittent lytic replication phases [15]. Both viral life cycles are characterized by distinct gene expression patterns, and importantly, both the latent and lytic gene products of gammaherpesviruses can contribute to pathogenesis [16]. Latency is characterized by minimal viral gene expression, allowing the virus to remain dormant within host cells, evading immune surveillance and thereby establishing long-term persistence. In contrast, the lytic replication phase is marked by extensive gene expression and active viral replication, resulting in the production and dissemination of infectious virions. Importantly, lytic oncogenic virus infection is often associated with inflammatory symptoms due to cell lysis and release of pro-inflammatory cytokines. In regions additionally burdened by a high prevalence of other communicable diseases presenting with inflammation, such as tuberculosis (TB) or (more recently) COVID-19, lytic EBV or KSHV infection may mimic and/or exacerbate these diseases, leading to misdiagnoses or late diagnoses [4].

Various environmental and viral stimuli are implicated in the transition from latency to lytic reactivation, including co-infections and associated inflammatory conditions [1]. Indeed, we have previously demonstrated that elevated blood KSHV viral load (VL), as a proxy for lytic reactivation, was a strong predictor of death in hospitalized HIV-infected patients presenting with TB symptoms [4]. More recently, we showed that KSHV co-infection was associated with mortality in hospitalised COVID-19 patients [17], while in non-hospitalised unvaccinated HIV-infected patients, repeated SARS-CoV-2 exposure led to reactivation of KSHV [5], particularly in patients with elevated EBV VL in the peripheral blood [18].

There is limited research on lytic oncogenic herpesvirus infection in SSA, which predominantly focuses on adult, HIV-infected individuals due to the increased risk of KSHV- or EBV-associated pathologies in this patient group [1]. Although transmission of both viruses mainly occurs during early childhood, data on KSHV and EBV seroprevalence and their infection dynamics in the context of co-infections presenting with inflammation in children remain scarce, even in highly endemic areas such as SSA.

Despite significant exposure to SARS-CoV-2, children have largely been protected from severe illness during the COVID-19 pandemic, implying that natural immunity may offer protection against serious disease outcomes [19]. However, in rare cases, severe postinfectious complications of SARS-CoV-2 infection can affect children and young adolescents. Multisystem Inflammatory Syndrome in Children (MIS-C) is the most severe acute inflammatory reaction to SARS-CoV-2. MIS-C typically presents 2–6 weeks after infection and is characterized by persistent fever, gastrointestinal symptoms, rash, conjunctivitis, and, in many cases, cardiovascular involvement, including myocardial dysfunction and coronary artery dilatation. Laboratory findings often reveal systemic hyperinflammation with markedly elevated C-reactive protein (CRP), ferritin, and D-dimer [20,21]. With a sixfold increased risk of developing MIS-C, children of African ancestry are disproportionately affected compared to Caucasian children [22,23]. Recently, EBV reactivation associated with the hyperinflammatory state seen in MIS-C was reported in children from Europe and South America [24]; however, African children were not included in this multi-centre study. Of note, MIS-C’s clinical manifestations overlap with Kawasaki disease (KD), an acute vasculitis of largely unknown etiology. KD typically arises independently of SARS-CoV-2 infection and is not associated with the same degree of systemic hyperinflammation or myocardial dysfunction [25]. In contrast to MIS-C, which occurs in children with a median age of approximately 9 years, KD predominantly affects those under 5 years of age [26].

Our previous research on adult patient cohorts provided strong clinical evidence that lytic KSHV infection mimicked and/or exacerbated pre-existing inflammatory conditions such as TB or COVID-19 [4,17]. Taking these observations into pediatric patients, we herein assessed both seroprevalence of EBV and KSHV and their infection dynamics in the context of MIS-C, KD, and non-specific inflammation in immunocompetent children aged 0–15 years from Cape Town, South Africa, recruited over the course of the COVID-19 pandemic.

## 2. Results

### 2.1. Demographic, General Clinical and Virological Characteristics of the Study Participants

This cross-sectional study consisted of 175 children, aged between 0 and 15 years old (median age 4.6 years), who presented at Red Cross War Memorial Children’s Hospital, a tertiary-level hospital that services the city of Cape Town, South Africa, and acts as a referral centre for a larger area within the province. It manages children with a full spectrum of illnesses and disorders. Patients were recruited between July 2020 (coinciding with the peak of the first COVID-19 wave in SA) and February 2024, covering all subsequent waves of the pandemic. All demographic and clinical characteristics are summarised in Table 1.

**Figure 1:**
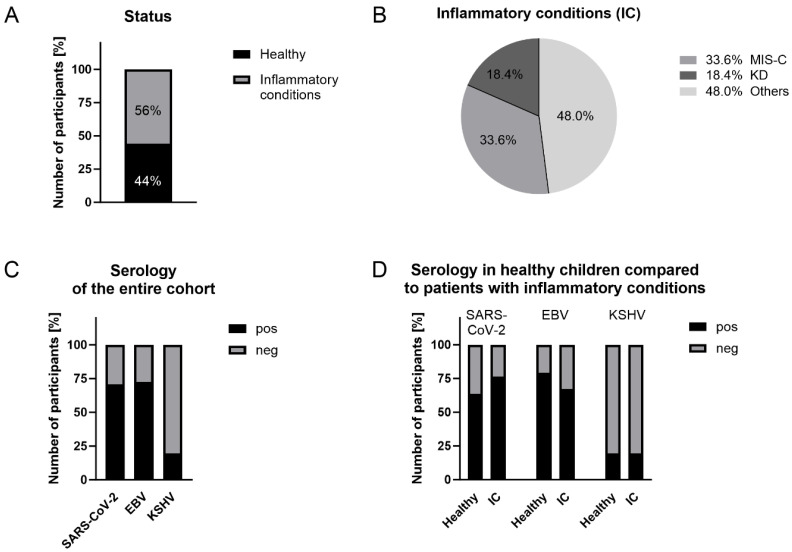
Overview of disease status and SARS-CoV-2/EBV/KSHV serology of all study participants (n = 175). (**A**) Percentage of study participants grouped into healthy and those with an inflammatory condition (IC), which was further defined as MIS-C, KD or others (**B**). (**C**) Percentage of participants testing seropositive for SARS-CoV-2, EBV or KSHV; (**D**) comparison of % seropositivity for SARS-CoV-2, EBV and KSHV between healthy children and patients presenting with IC.

All, except one patient, were HIV negative and had not been vaccinated against SARS-CoV-2. 68 (38.9%) individuals were female and 101 (57.7%) were male, while the sex of 6 individuals (3.4%) was not recorded. The racial and ethnic distribution of the study participants consisted of black and mixed-race children, with 79 (46.7%) being of Black African ancestry, 90 (53.3%) being of mixed ancestry, and 6 individuals (3.4%) having no recorded ethnicity. More than half of the patients (56%) had inflammatory conditions (IC). After physical examination and diagnostic testing, 33 (33.7%) of those were clinically diagnosed with MIS-C and 18 (18.4%) were diagnosed with KD. Children with diverse diagnoses are referred to as “other inflammatory conditions” (“others”) (Supplementary Table S1). The remaining 77 children (44%) of the study participants were healthy individuals, representing a control group (Figure 1A,B).

When assessing SARS-CoV-2 seroprevalence, we found that the majority of the study participants (70.9%) tested positive for S1 and RBD SARS-CoV-2 antibodies in the absence of COVID-19 vaccination. Based on EBV-EBNA1-specific serology, 72.6% of children were EBV-positive, which is consistent with the ubiquitous EBV distribution worldwide. In contrast, only 19.4% of the children tested positive for either KSHV-specific LANA or K8.1 antibodies (Figure 1C), which is considerably lower compared to our previous studies on adult HIV-infected patients from the same geographic region, where we observed seroprevalence of up to 53.5% [5]. Of note, the herein applied serology tests are indicative of previous exposure to the respective viruses but do not allow conclusions about viral activity.

We further assessed the distribution of SARS-CoV-2, EBV, and KSHV serology in healthy individuals versus the IC group. Seroprevalence of SARS-CoV-2 was 63.6% in the healthy group and slightly higher in the IC group (76.5%) (*p* = 0.07) (Figure 1D, Table 2). While we observed a trend of higher EBV-seropositivity (79.2%) in healthy children compared to the IC group (67.3%) (*p* = 0.08), positive responses to KSHV antigens were very similar between the two groups, with 19.7% and 20.0%, respectively (Figure 1D, Table 2). Taken together, we observed almost similar distributions of seropositivity to each virus between healthy individuals and patients with IC.

However, we found differences in the magnitude of serological responses to EBV and SARS-CoV-2 between the groups: median OD values against EBV EBNA1 were lower in children with IC (median OD 4.21, IQR: 0.38–5.23) compared to healthy children (median OD 4.90, IQR (2.30–5.44), *p* = 0.026, Table 2). In contrast, higher antibody titres against both SARS-CoV-2 antigens were observed in the IC group compared to healthy children. For RBD, we observed a median OD of 6.31 (IQR: 3.52–9.24) in the IC group versus 3.41 (IQR: 1.96–6.83) in healthy children, *p* < 0.001, Table 2. Similarly, S1 antibody titres showed a median OD of 7.82 in the IC group (IQR: 3.36–11.69) compared to 4.76 (IQR: 1.59–8.41) in healthy children, *p* = 0.013, Table 2. As children with MIS-C were enrolled on the basis of a COVID-19-related diagnosis, elevated antibody titres in this subgroup were anticipated, which likely contributed to the higher overall values in the IC group.

The univariate analysis on demographic variables further revealed differences in the distribution of gender (*p* = 0.032) and ethnicity (*p* = 0.041) between the two groups (Table 2). In the healthy group, 68.9% of children were male and 31.1% were female, whereas in the IC group, 52.6% were male and 47.4% were female. Thus, there is a higher proportion of female children in the IC group. We further observed a higher proportion of children of Black African ancestry (53.7%) in the IC group compared to 37.8% in the healthy group. The proportion of children of mixed ancestry was higher in the healthy group (62.2%) compared to the disease group (46.3%). These differences are in line with previously reported hospital referral patterns [22].

### 2.2. Assessment of Detectable KSHV and EBV DNA in the Peripheral Blood

Both inflammation and SARS-CoV-2 co-infection are implicated in KSHV and EBV lytic reactivation [1,5,27,28,29]. As one of our recent clinical studies showed significantly higher odds of KSHV reactivation in adult HIV-infected patients who were previously exposed to SARS-CoV-2 [5], particularly in the context of elevated EBV VL [18], we assessed whether similar infection dynamics may also occur in paediatric patients.

We first screened all KSHV-seropositive children for KSHV VL (indicating KSHV reactivation, though not excluding primary infection or accumulation of latently infected B lymphocytes) by performing quantitative real-time PCR. In contrast to our previous findings in adult HIV-infected patients, we did not detect KSHV VL in any of the KSHV-seropositive children.

**Figure 2:**
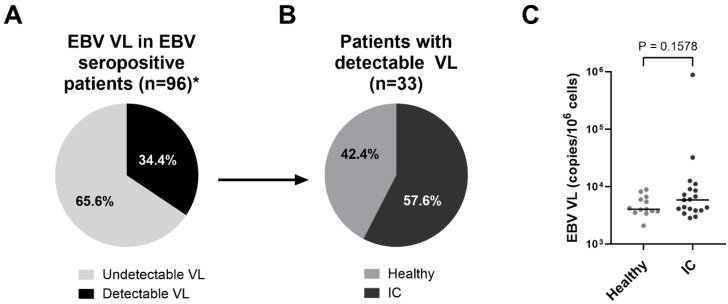
Distribution and quantification of detectable EBV VL. (**A**) Proportion of EBV-seropositive individuals with detectable versus undetectable EBV VL. (**B**) Distribution of participants with detectable EBV VL into healthy controls and patients with IC. (**C**) EBV copy numbers in healthy controls and patients with IC. Median values are indicated by solid lines. Statistical significance between the groups was assessed using the Mann–Whitney U test. * Insufficient sample volume for 31 of the EBV seropositive patients (n = 127).

In addition, all EBV-seropositive participants were assessed for EBV VL. Detectable VL was observed in 34.4% of seropositive individuals, among whom 42.4% were healthy controls and 57.6% had an IC (Figure 2A,B). There was no difference in median EBV VL observed in IC patients, with a median of 5.85 × 10^3^ copies/10^6^ cells compared to 4.04 × 10^3^ copies/10^6^ cells in healthy controls (*p* = 0.1578) (Figure 2C). One IC patient exhibited highly elevated EBV VL (8.8 × 10^5^ copies/10^6^ cells), which corresponded to the only HIV-positive individual among all study participants.

### 2.3. Impact of SARS-CoV-2 and EBV Infection Status on Inflammatory Presentation

Following the univariate analysis, which identified differences in the magnitude of EBV and SARS-CoV-2 immune responses between healthy children and children presenting with inflammation (Table 2), we performed a binomial logistic regression to confirm these observations under adjusted conditions by controlling for sex, ethnicity, and age (Table 3). Using this model, we observed a difference in the RBD antibody titres (*p* = 0.001) between healthy children and those presenting with inflammation, whereas EBNA1 antibody titres were no longer significantly associated with the inflammatory status after adjustment. In the logistic regression model assessing RBD antibody titres, sex and ethnicity remained significant predictors, while EBNA1 antibody titres were not independently associated with the outcome after adjustment (Table 3).

**Figure 3:**
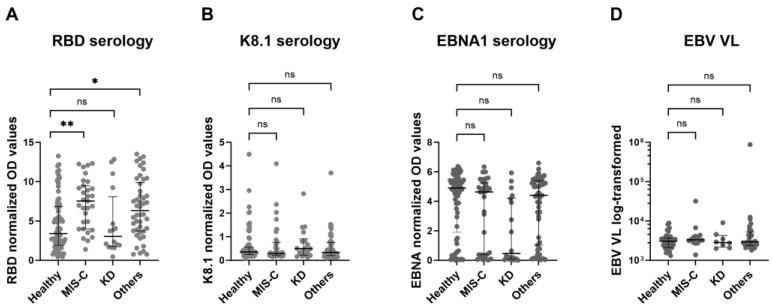
Comparison of SARS-CoV-2, KSHV and EBV serology, and EBV VL in the different IC groups and healthy children. Normalized OD values for RBD, EBNA1 and K8.1 were plotted for each group. EBV VL is depicted as log-transformed values (**D**). As data were not normally distributed in all groups (determined by Shapiro–Wilk test), medians with IQR were plotted. Analysis of covariance (ANCOVA), with group included as a fixed factor and age included as a covariate was performed to determine significant differences in antibody titre (**A**–**C**) or EBV VL (**D**) between the groups. Significant differences are indicated with * (*p* > 0.05), ** (*p* < 0.01).

Following on from these results, we were interested in whether the antibody titres against SARS-CoV-2 (RBD), EBV (EBNA1) and KSHV (K8.1) differed between the three IC groups, i.e., MIS-C, KD, and other diverse IC, compared to the healthy control group. Indeed, we found that SARS-CoV-2 RBD values were higher in children with either MIS-C or other IC (*p* < 0.01), but not in children with KD disease, in comparison to healthy children (Figure 3A).

No differences for EBV (EBNA-1) and KSHV (K8.1) serology between the different groups were identified (Figure 3B,C), indicating that immune responses to EBV and KSHV were not influenced by the inflammatory conditions analysed in these patients. Likewise, no differences in EBV VL were observed between the groups (Figure 3D).

### 2.4. Age-Dependent SARS-CoV-2, EBV and KSHV Transmission Dynamics

We further aimed to assess transmission dynamics of SARS-CoV-2, EBV, and KSHV among the study participants and to assess the association between age and risk of infection. While the recruited patients comprised children between 0 and 15 years, the majority of the 175 participants were under the age of 7 (64%), and 36% were between 7 and 15 years old.

Logistic regression analysis for infection risk with SARS-CoV-2 with age (controlling for the older group of MIS-C patients as a confounding factor) indicated increasing odds of becoming SARS-CoV-2 seropositive with each additional year of age (OR 1.118; *p* = 0.019), Table 4.

Whilst no association was found between KSHV seropositivity and age, we observed increased odds of being seropositive for EBV with every year of age (OR 1.352; *p* < 0.001) (Table 4). For all EBV-seropositive patients, we further assessed the association of age and EBV VL (excluding the highly elevated EBV VL outlier coinciding with the only HIV-positive patient among all study participants; see Figure 2C) and observed that the odds of detectable EBV VL decreased by 15.9% with every year of age (OR 0.738, *p* = 0.01) (Table 4).

**Figure 4:**
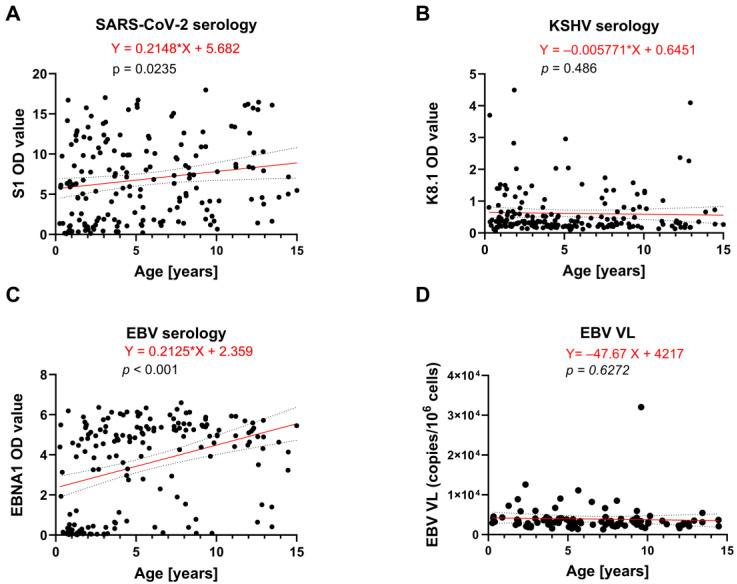
Linear regression analysis of the association between age and SARS-CoV-2, KSHV and EBV serology, and EBV VL. Antibody titres represented by normalized OD values of ELISA assays for (**A**) SARS-CoV-2 S1, (**B**) KSHV K8.1 and (**C**) EBV-EBNA1 are plotted against age. (**D**) EBV VL, assessed for all EBV seropositive patients but excluding the outlier coinciding with HIV positivity, is represented as copy numbers per million cells. Trend lines of linear regression are indicated in red and are fitted straight. The grey dotted lines indicate 95% confidence intervals. Results are considered statistically significant when *p* < 0.05.

We next assessed the association of age with immune responses against SARS-CoV-2, KSHV, and EBV as continuous variables using OD values in a linear regression (Figure 4A–C). Consistent with the results of the logistic regression analysis (Table 4), no age-related increase was observed for KSHV antibody titres against K8.1 (Figure 4B). In contrast, we observed increases for both SARS-CoV-2 (S1) and EBV (EBNA-1) antibody titres with age (*p* = 0.0235 and *p* < 0.001, respectively), Figure 4A,C.

As logistic regression analysis indicated a significantly lower risk of detectable EBV VL with advancing age (Table 4), we finally investigated whether VL levels varied across age using a linear regression. While older individuals were less likely to have detectable EBV VL (Table 4), VL levels among those who were positive remained independent of age (Figure 4D).

## 3. Discussion

Although it has been well established that gammaherpesvirus transmission in SSA primarily occurs in childhood from mother to child and/or between siblings [10,30,31,32,33,34], with reported increased infection risk in the context of HIV co-infection [30,32], there are limited studies on the infection dynamics of these viruses in paediatric cohorts. Reactivation of EBV and KSHV from latency has been mostly described in adult patients and with a focus on co-infections causing inflammation (reviewed in [1]). In the context of the recent COVID-19 pandemic, we and others have reported on increased EBV and/or KSHV viral DNA in the peripheral blood (as a proxy for lytic infection) in adult patients exposed to SARS-CoV-2 and/or hospitalized with COVID-19 [5,17,35,36]. While this interpretation is based on the notion that primary infection primarily occurs in childhood, followed by the establishment of latency in the host, PCR-based detection of viral DNA in the peripheral blood of adult patients below a certain threshold could also reflect an accumulation of latently infected circulating cells.

Among the herein reported paediatric patients recruited during the COVID-19 pandemic in the Western Cape, South Africa, we found high SARS-CoV-2 seroprevalence (70.9%) in the absence of vaccination and a substantially higher number of EBV-seropositive than KSHV-seropositive children (72.6% versus 19.4%), with only EBV seropositivity significantly increasing with age. These differences in paediatric gammaherpesvirus age-dependent seroprevalence support earlier studies that reported ubiquitous EBV infection but low, stable paediatric KSHV seroprevalence (7–9%) in KwaZulu Natal, South Africa [34,37], which was notably lower than in endemic areas like Uganda, where seropositivity among children rises sharply with age [34]. Of note, gammaherpesvirus seroprevalence among our study participants was lower than in adult patients recruited from the same geographic area, where we reported 30–50% seropositivity for KSHV [4,5,17] and >90% for EBV [18]. While age may increase the likelihood of gammaherpesvirus infection, the underlying HIV infection in our previously described adult cohorts may play a major role in viral acquisition [38,39]. This age-related increase in gammaherpesvirus infection most likely reflects a combined exposure risk through additional transmission routes such as sexual contact, medical procedures, or blood transfusions [11,12,13,14].

While about a third of the EBV-seropositive children in our study displayed detectable EBV DNA in the peripheral blood, none of the KSHV-seropositive children showed active KSHV infection. There was a slight trend of higher EBV VL in children presenting with inflammation, with one patient displaying particularly elevated EBV VL in the context of their HIV co-infection. Interestingly though, the presence of detectable EBV DNA decreased with age when assessed in the context of all study participants, suggesting robust immune control in the absence of HIV infection in all but one patient, despite inflammation as a potential trigger for lytic reactivation.

We could neither link prior EBV and/or KSHV infection nor elevated EBV VL to a specific inflammatory group. This is importantly in contrast with a recent multi-centre study including children from Europe and South America, which reported EBV reactivation associated with the hyperinflammatory state seen in MIS-C, reportedly driven by TGFβ-induced immune suppression [24]. In that study, children with MIS-C had a higher rate of EBV seropositivity (around 80%) compared to age-matched healthy controls (about 50%), suggesting that prior EBV infection is a major risk factor for developing MIS-C following SARS-CoV-2 infection. Our current data are not able to confirm this link between EBV and MIS-C.

Geographic origin, ethnicity and the complexity of immune responses to co-infection(s) may play important contributing roles in gammaherpesvirus infection dynamics. Although some of our paediatric patients presented with severe inflammatory symptoms (indicative of MIS-C and KD), all but one were HIV-negative who may have well-preserved immune functions to control latent gammaherpesvirus infection. While our study revealed only slightly increased EBV DNA levels in patients presenting with an inflammatory condition, previous studies reported on significantly elevated EBV VL in HIV-exposed uninfected infants presenting with serious adverse clinical events, including malaria and pneumonia, in Uganda [31], and EBV viraemia associated with acute febrile illnesses in Kenyan children from malaria endemic areas [40]. None of the paediatric patients in our study displayed lytic KSHV infection; indeed, only a limited number of studies have reported on KSHV viraemia in children, primarily from endemic regions and in the context of HIV co-infection and Kaposi’s sarcoma [41,42].

## 4. Materials and Methods

### 4.1. Study Participants and Ethics

A total of 175 patients previously recruited from Red Cross War Memorial Children’s Hospital, Cape Town, South Africa, between July 2020 and February 2024 were enrolled into the present study. The Paediatric Rheumatology Research group runs a biorepository that recruits a wide variety of children with inflammatory and immune diseases in addition to healthy children undergoing elective surgery for non-inflammatory indications (e.g., plastic surgery, circumcisions, squint repair). A subset of healthy children and children diagnosed with KD (as per AHA criteria [43]), MIS-C (as per CDC criteria [44]), and “other inflammatory illnesses” (as diagnosed by the treating physician, Supplementary Table S1) were chosen for inclusion into the current study if there were sufficient samples available for the assays performed. Both female and male children were included in the study and were aged between 0 and 15 years old, HIV-negative (except one participant), and had not been vaccinated against SARS-CoV-2. Brief demographical and clinical information is listed in Table 1**.** The clinical features, therapy, and outcomes of the study participants have been previously described [22,45]. Ethics approval was obtained from the human research ethical committee (HREC) at the Health Sciences Faculty of the University of Cape Town (UCT). This study (HREC 400/2024) represents a retrospective observational sub-study of the biorepository study (HREC 599/2020; HREC 112/2012). All participants were recruited with consent.

### 4.2. Sample Preparation

Plasma was removed from whole blood samples and used in serological assays. Genomic DNA was subsequently isolated from the remaining leukocyte-enriched buffy coat and erythrocyte fraction using the QIAmp Blood Midi Kit (Qiagen, Hilden, Germany) according to the manufacturer’s instructions. Quantification and quality assessment of the isolated genomic DNA were performed by a NanoDrop One/Onec Spectrophotometer (Thermo Fisher Scientific, Waltham, MA, USA).

### 4.3. SARS-CoV-2 Serology

SARS-CoV-2 serology was determined by an in-house enzyme-linked immunosorbent assay (ELISA) against RBD and S1 proteins [46], using a protocol adapted from Makatsa et al. [47]. OD values of all samples were normalized to the average (+2 SD) of pre-pandemic samples. Samples were considered positive if normalized OD values for both S1 and RBD antigens were above the cut-off, which was set as 2.

### 4.4. EBV Serology

EBV serology was determined by quantifying concentrations of Epstein-Barr virus nuclear antigen-1 (EBNA-1) using the EBNA-1 IgG-ELISA kit (Abnova, Taipei City, Taiwan) following the manufacturer’s instructions. Results of absorbance readings were calculated following the manufacturer’s protocol, where samples and standards were normalized to the value of the negative control. The cut-off value was determined according to the manufacturer’s instructions by multiplying the OD value of one provided standard (“Standard D”) by the correction factor provided in the QC certificate. All readings with a value above 1.0 were considered positive.

### 4.5. KSHV Serology

To determine KSHV seroprevalence, ELISA assays against the lytic structural glycoprotein K8.1 and the latency-associated nuclear antigen (LANA/ORF73) were performed using pre-coated plates kindly provided by Dr Denise Whitby (National Cancer Institute (NCI) at Frederick, National Institute of Health (NIH), Frederick, MD, USA). Previously determined equations were used to set cut-off OD values for the K8.1 and LANA ELISA, respectively [48]. Samples above the calculated cut-off for either K8.1 or LANA, or both, were considered as positive.

### 4.6. KSHV and EBV Viral Load Assays

VL assays for KSHV and EBV were performed using DNA extracted from blood samples of KSHV- and/or EBV-seropositive individuals. Quantitative TaqMan PCR targeting the lytic KSHV K6 gene and the EBV polymerase gene, respectively, was carried out as previously described [49,50]. To control for input DNA and determine cellular equivalents, KSHV and EBV copy numbers were normalized to the human endogenous retrovirus 3 (ERV-3) gene [51].

Each 50 µL PCR reaction contained 100 pmol forward and reverse primers, 5 pmol FAM/TAMRA-labelled probe, 250 ng DNA template, and 2× Universal Master Mix (Thermo Fisher Scientific, Waltham, MA, USA). Quantification of KSHV, EBV, and ERV-3 DNA was performed using standard curves generated from plasmids containing K6, EBV polymerase, and ERV-3 sequences, respectively [40,41]. All standards and samples were analysed in triplicate. Amplification was performed on a Roche LightCycler 480II (Roche Diagnostics Ltd., Rotkreuz, Switzerland) under the following cycling conditions: 50 °C for 2 min, 95 °C for 8 min, followed by 45 cycles of 95 °C for 15 s and 60 °C for 1 min.

For normalization, average viral copy numbers per sample were divided by ERV-3 copy numbers, assuming two copies per diploid genome, and expressed as viral copies per 10^6^ cells. The assay cut-off was defined as a cycle threshold (Ct) value ≤ 35, with samples above this threshold considered negative.

### 4.7. Statistical Analysis of Study Data

Statistical analysis of data was performed using SPSS version 29.0.0.0 (IBM Corp, Armonk, NY, USA) and GraphPad Prism version 10.1.2 (GraphPad Software, LLC, San Diego, CA, USA).

For general demographic and virological characteristics of the study participants, data were either presented as numbers and percentages or medians with interquartile range (IQR). To assess the association between age and disease, linear regression was performed for continuous dependent variables, and trendlines were fitted as straight lines. For categorical dependent variables, and to further control for confounding factors when assessing associations of disease status and contributing factors, a binomial logistic regression was performed. Univariate analyses were performed using the chi-square test for categorical variables and the Mann–Whitney U test for scale variables. To assess normality of data, the Shapiro–Wilk test was performed. If data were not normally distributed, median values with IQR were plotted. Analysis of covariance (ANCOVA), with group included as a fixed factor and age included as a covariate to account for potential confounding by age, was used to determine statistical significance of differences between multiple conditions. Where applicable, pairwise comparisons of adjusted group means were performed using Bonferroni correction for multiple testing. *p*-values were two-tailed and considered significant if *p* < 0.05. Graphical data presentation was created using GraphPad Prism (version 10.1.2).

## 5. Conclusions

In summary, the herein reported paediatric gammaherpesvirus infection dynamics are strikingly different from previously reported adult cohorts who were mainly investigated in the context of HIV co-infection. Inflammatory conditions seem to only marginally affect underlying latent EBV infection but have no impact on KSHV infection dynamics. Further longitudinal clinical studies with a larger number of patients and with balanced representatives from all gender and ethnicity groups would need to be conducted to substantiate our findings on this rather small group of individuals and to identify temporal relations between inflammatory disease onset and gammaherpesvirus lytic infection.

## Figures and Tables

**Table 1:** General demographic and virological characteristics of the study participants (n = 175). Data are either presented as numbers and percentages or median with interquartile range (IQR).

Characteristic		n (%) or Median (IQR)
Sex	Female Male Unknown	68 (38.9%) 101 (57.7%) 6 (3.4%)
Age, years		4.6 (2.0–8.3)
Ethnicity	Black African Mixed Ancestry Not recorded	79 (46.7%) 90 (53.3%) 6 (3.4%)
HIV status	Negative Positive	174 (99.4%) 1 (0.6%)
Healthy		77 (44%)
Inflammatory conditions (IC)	All MIS-C KD Others	98 (56%) 33 (33.6% of IC) 18 (18.4% of IC) 47 (48.0% of IC)
KSHV serology	Negative Positive	141 (80.6%) 34 (19.4%)
KSHV VL in KSHV-seropositive samples	Not detectable Detectable	34 (100%) 0 (0%)
EBV serology	Negative Positive	48 (27.4%) 127 (72.6%)
EBV VL in EBV-seropositive samples	Not detectable Detectable	63 (65.6%) 33 (34.4%)
SARS-CoV-2 serology	Negative Positive	51 (29.1%) 124 (70.9%)
SARS-CoV-2 vaccinated	No Yes	175 (100%) 0 (0%)

**Table 3:** Logistic regression of factors assessed for IC in all study participants (n = 175). Results are statistically significant if *p* < 0.05 which is indicated in bold.

Variable	Unadjusted Odds Ratio	95% CI for Unadjusted Odds Ratio		Adjusted Odds Ratio	95% CI for Adjusted Odds Ratio		*p*-Value
		Lower	Upper		Lower	Upper	
**RBD** norm. OD	1.151	1.054	1.255	1.166	1.061	1.281	**0.001**
Sex	0.501	0.265	0.947	0.478	0.244	0.940	**0.032**
Ethnicity	0.525	0.283	0.975	0.522	0.271	1.003	**0.051**
Age	0.953	0.883	1.028	0.947	0.872	1.028	**0.193**
**EBNA1** norm. OD	0.861	0.752	0.986	0.876	0.752	1.021	**0.089**
Sex	0.501	0.265	0.947	0.537	0.279	1.033	0.063
Ethnicity	0.525	0.283	0.975	0.544	0.288	1.0	0.061
Age	0.953	0.883	1.028	0.985	0.906	1.071	0.729

**Table 4:** Logistic regression to assess association of age [years] and seropositivity for SARS-CoV-2, KSHV and EBV among all study participants (n = 175) and EBV VL in EBV seropositive patients (n = 97), excluding the only HIV-positive participant. Association of age and SARS-CoV-2 serology was corrected for MIS-C as disease groups. Results are statistically significant if *p* < 0.05 which is indicated in bold.

Variable	Unadjusted Odds Ratio	95% CI for Unadjusted Odds Ratio		*p*-Value
		Lower	Upper	
SARS-CoV-2	1.118	1.018	1.227	**0.019**
KSHV	0.966	0.876	1.065	0.486
EBV	1.352	1.187	1.541	**<0.001**
EBV VL	0.841	0.738	0.959	**0.010**

**Table 2:** Univariate analysis of clinical and demographic variables. Data are either presented as numbers and percentages or median with interquartile range (IQR). A chi-square test was performed for categorical variables; scale variables were analysed using the Mann–Whitney U test. Statistically significant results (*p* < 0.05) are indicated in bold. Participants with missing data are excluded per characteristic.

Variable		Healthy n (%) or Median (IQR)	IC n (%) or Median (IQR)	*p*-Value
KSHV serology	Negative Positive	61 (80.3%) 15 (19.7%)	76 (80.0%) 19 (20.0%)	0.988
K8.1 serology	Negative Positive	65 (84.4%) 12 (15.6%)	79 (80.6%) 19 (19.4%)	0.513
LANA serology	Negative Positive	72 (93.5%) 5 (6.5%)	95 (96.9%) 3 (3.1%)	0.281
SARS-CoV-2 serology	Negative Positive	28 (36.4%) 49 (63.6%)	23 (23.5%) 75 (76.5%)	0.069
EBV (EBNA1) serology	Negative Positive	16 (20.8%) 61 (79.2%)	32 (32.7%) 66 (67.3%)	0.081
KSHV K8.1 normalized OD		0.35 (0.25–0.54)	0.33 (0.21–0.78)	0.730
KSHV LANA normalized OD		0.17 (0.12–0.32)	0.16 (0.12–0.26)	0.767
SARS-CoV-2 RBD normalized OD		3.41 (1.96–6.83)	6.31 (3.52–9.24)	**<0.001**
SARS-CoV-2 S1 normalized OD		4.76 (1.59–8.41)	7.82 (3.36–11.69)	**0.013**
EBNA1 normalized OD		4.90 (2.30–5.44)	4.21 (0.38–5.23)	**0.026**
EBV VL *	Undetectable Detectable	31 (68.9%) 14 (31.1%)	32 (62.7%) 19 (37.3%)	0.527
Ethnicity	Black Mixed Ancestry	28 (37.8%) 46 (62.2%)	51 (53.7%) 44 (46.3%)	**0.041**
Sex	Female Male	23 (31.1%) 51 (68.9%)	45 (47.4%) 50 (52.6%)	**0.032**
Age		5.08 (2.49–9.05)	4.42 (1.76–8.10)	0.183

* Only EBV seropositive participants were assessed for EBV VL, of whom 31 had insufficient sample volume.

## Data Availability

The data that support the findings of this article are openly available at PubMed (https://pubmed.ncbi.nlm.nih.gov/) (accessed on 12 December 2025).

## Supplementary Materials

The following supporting information can be downloaded at: https://www.mdpi.com/article/10.3390/ijms27031275/s1.
