# Supplementary material for: Assessing Seroprevalence and Infection Dynamics of Oncogenic Gammaherpesviruses in South African Paediatric Patients Presenting with Inflammatory Conditions
Source: Int J Mol Sci. 2026 Jan 27;27(3):1275. doi: 10.3390/ijms27031275 (PMC12898391; doi:10.3390/ijms27031275)
Supplement: Supplementary file 1 [file ijms-27-01275-s001.zip › ijms-4069035-supplementary.pdf]

**Supplementary Table S1:** Summary of the patient group presenting with "other inflammatory conditions" (n=47).

| Diagnosis | Number of patients |
|---|---|
| Gastroenteritis | 8 |
| Dysentery | 5 |
| Upper respiratory tract infection (URTI) | 5 |
| Unknown | 2 |
| Sepsis | 2 |
| Juvenile idiopathic arthritis (JIA) | 2 |
| Adenovirus | 2 |
| Juvenile dermatomyositis (JDM) | 2 |
| Pneumonia | 2 |
| Tuberculosis | 1 |
| Appendicitis | 1 |
| Typhoid | 1 |
| Adenoviral myocarditis | 1 |
| Salmonella | 1 |
| Pyrexial illness with no organism found, responded to antibiotics | 1 |
| Acute generalized exanthematous pustulosis (AEGP) | 1 |
| Toxic shock syndrome | 1 |
| Scurvy | 1 |
| Mumps | 1 |
| Rheumatic fever | 1 |
| Lower respiratory tract infection (LRTI) | 1 |
| Viral meningitis | 1 |
| Toxic epidermonecrolysis | 1 |
| Erythema multiforme secondary to HSV-1 | 1 |
| Enteroviral encephalitis | 1 |
| Tonsilitis | 1 |
